# Anthracycline-induced cardiotoxicity: From pathobiology to identification of molecular targets for nuclear imaging

**DOI:** 10.3389/fcvm.2022.919719

**Published:** 2022-08-03

**Authors:** Jeremy Jong, James R. Pinney, René R. Sevag Packard

**Affiliations:** ^1^Department of Medicine, Division of Cardiology, David Geffen School of Medicine, University of California, Los Angeles, Los Angeles, CA, United States; ^2^Ronald Reagan UCLA Medical Center, Los Angeles, CA, United States; ^3^Veterans Affairs West Los Angeles Medical Center, Los Angeles, CA, United States; ^4^Department of Physiology, David Geffen School of Medicine, University of California, Los Angeles, Los Angeles, CA, United States; ^5^Jonsson Comprehensive Cancer Center, University of California, Los Angeles, Los Angeles, CA, United States; ^6^Molecular Biology Institute, University of California, Los Angeles, Los Angeles, CA, United States; ^7^California NanoSystems Institute, University of California, Los Angeles, Los Angeles, CA, United States

**Keywords:** anthracycline, cardiotoxicity, molecular imaging, nuclear medicine, clinical application

## Abstract

Anthracyclines are a widely used class of chemotherapy in pediatric and adult cancers, however, their use is hampered by the development of cardiotoxic side-effects and ensuing complications, primarily heart failure. Clinically used imaging modalities to screen for cardiotoxicity are mostly echocardiography and occasionally cardiac magnetic resonance imaging. However, the assessment of diastolic and global or segmental systolic function may not be sensitive to detect subclinical or early stages of cardiotoxicity. Multiple studies have scrutinized molecular nuclear imaging strategies to improve the detection of anthracycline-induced cardiotoxicity. Anthracyclines can activate all forms of cell death in cardiomyocytes. Injury mechanisms associated with anthracycline usage include apoptosis, necrosis, autophagy, ferroptosis, pyroptosis, reactive oxygen species, mitochondrial dysfunction, as well as cardiac fibrosis and perturbation in sympathetic drive and myocardial blood flow; some of which have been targeted using nuclear probes. This review retraces the pathobiology of anthracycline-induced cardiac injury, details the evidence to date supporting a molecular nuclear imaging strategy, explores disease mechanisms which have not yet been targeted, and proposes a clinical strategy incorporating molecular imaging to improve patient management.

## Introduction

Anthracyclines are one of the most commonly prescribed chemotherapies and are used to treat a variety of cancers. Although effective agents, their benefits are sometimes compromised by acute and/or late-onset cardiotoxic side effects. A study that compared adult survivors of pediatric cancer with their siblings found that survivors (that had been treated with anthracyclines and/or radiotherapy) had a 15-fold higher risk of developing heart failure (1). The risk of subsequent heart failure in pediatric patients treated with anthracyclines was demonstrated to be highly dose-dependent, particularly in cumulative anthracycline doses ≥300 mg/m^2^, and to increase over time (3.3% at 2 years, 4.5% at 10 years, and 9.8% at 20 years after the first dose) (2, 3). Similarly, a retrospective analysis of three phase III clinical trials with adult patients indicated that treatment with a cumulative doxorubicin dose of ≥400 mg/m^2^ led to a 5% incidence of heart failure, rising to up to 26% at a cumulative dose of 550 mg/m^2^ (4). It must be emphasized, however, that (i) no “safe dose” of anthracyclines truly exists, (ii) late effects leading to heart failure can occur and need to be monitored, and (iii) risk assessment needs to be individualized with a particular focus on pre-existing heart disease and/or cardiovascular risk factors such as hypertension. Thus, given the wide individual variability in patient risk of developing anthracycline-induced cardiac injury, risk stratification must be done on a case-by-case basis.

In clinical practice, side effects of anthracyclines are balanced by limiting the dosage while tightly monitoring for clinical manifestations of cardiotoxicity. Typically, imaging modalities such as echocardiography are used to monitor for cardiotoxicity, commonly defined as a decline in LVEF of ≥10% to a final value <50% (5). A recent International Cardio-Oncology Society (IC-OS) consensus statement (6) proposed a standardized and more nuanced definition of cancer therapy-related cardiac dysfunction (CTRCD), applicable to anthracyclines, as follows: (1) Asymptomatic CTRCD graded as (i) mild (LVEF ≥50% and new decline in GLS >15% from baseline, and/or new rise in troponin I/T, BNP, NT-proBNP), (ii) moderate (new LVEF reduction by ≥10 percentage points to a LVEF 40–49%, or new LVEF reduction by <10 percentage points to a LVEF 40–49% and new decline in GLS >15% from baseline, and/or new rise in troponin I/T, BNP, NT-proBNP), and (iii) severe (new LVEF reduction to <40%). (2) Symptomatic CTRCD with supportive LVEF and diagnostic biomarkers, graded from mild to very severe based on heart failure symptoms, requirement for intensification of heart failure treatment, hospitalization, and/or inotropic or mechanical circulatory support.

However, these prognostic/diagnostic tools have several limitations, including their variability, lack of sensitivity, and inadequate detection of toxicity at a subclinical level. One of the main issues is that these parameters detect cardiotoxicity when the myocardium is already damaged and/or cardiac function impaired, which may hinder treatment options. Indeed, histological biopsy samples of patients that underwent doxorubicin treatment demonstrated that the myocardium could incur significant injury despite patients having a preserved/normal LVEF (7).

Therapeutic treatment for AIC remains limited. One of the most well studied agents, dexrazoxane, has been shown to significantly reduce cardiotoxicity in adults and pediatric patients when concurrently prescribed with anthracyclines (8–10). Recent studies have demonstrated the safety of dexrazoxane as a cardioprotective agent and confirmed its lack of interference with the anti-tumor action of anthracyclines. Indeed, an analysis of three Children’s Oncology Group (COG) trials that had randomized patients to doxorubicin with or without dexrazoxane (dexrazoxane:doxorubicin dose ratio 10:1, cumulative protocol-specified doxorubicin dose 100–360 mg/m^2^) with a median follow-up of 12.6 years demonstrated that dexrazoxane does not compromise long-term survival, and is not associated with mortality from acute myeloid leukemia or cardiovascular causes (11). This was followed by another COG analysis of four trials with pediatric patients that similarly were treated with doxorubicin with or without dexrazoxane (other than one trial in which all patients were assigned to dexrazoxane upfront), and with a median follow-up of close to 20 years, indicating that dexrazoxane does not negatively affect long-term mortality or second cancer risk (12). Angiotensin-converting enzyme inhibitors and beta-blockers have also been studied. In a study of 201 patients with AIC, Cardinale et al. observed that 64% of patients treated early (i.e., 1–2 months after completion of chemotherapy) with enalapril/carvedilol had complete LVEF recovery, while 0% of the patients treated 6 months post-chemotherapy had complete LVEF recovery (13). Whereas these findings were not corroborated in the prospective CECCY trial which randomized 200 HER2-negative breast cancer patients to carvedilol vs. placebo synchronous with anthracycline initiation (total 240 mg/m^2^ over 4 cycles), a benefit of betablockade was noted on the development of diastolic dysfunction (14).

These clinical findings underscore the importance of early disease detection and emphasize the need for additional methods to diagnose subclinical AIC. A promising such strategy is nuclear imaging that can map molecular processes perturbed in AIC using radioactively labeled probes (Table 1). Advancements in nuclear imaging have rendered imaging of pathological processes such as mitochondrial dysfunction, sympathetic innervation, and fibrosis, possible. With the ongoing dissection of the pathobiology of AIC at the molecular level, we predict these advances will permit the identification of novel molecular imaging targets and posit a future role for nuclear imaging that will be complementary to that of echocardiography (and/or cardiac MRI). The present review retraces the preclinical and clinical evidence supporting the use of a nuclear molecular imaging strategy in AIC, and offers new avenues for tracer development targeting injury pathways that have not yet been explored.

**TABLE 1:** Radiotracer, mechanism of uptake, and application for nuclear imaging of anthracycline-induced cardiotoxicity.

Radiotracer	Modality	Target	Cardiovascular application	Preclinical studies	Clinical studies
^18^F-FDG*	PET	Glucose transporters	Glucose metabolism	32	34–36,38
^11^C-Acetate	PET	Monocarboxylate transporter	Oxidative metabolism	43	45
^11^C-Acetoacetate	PET	Monocarboxylate transporter	Ketone body metabolism	44	
^18^F-DHMT	PET	Reactive oxygen species	Cytotoxicity	59	
^99m^Tc-Sestamibi*	SPECT	Mitochondrial membrane potential	Cytotoxicity/perfusion	51	
^68^Ga-Galmydar	PET	Mitochondrial membrane potential	Cytotoxicity/perfusion	52	
^18^F-MitoPhos	PET	Mitochondrial membrane potential	Cytotoxicity/perfusion	53	
^99m^Tc-Annexin V	SPECT	Externalized phosphatidylserine	Apoptosis	76	
^18^F-CP18	PET	Caspase-3 activity	Apoptosis	83	
^111^In-Antimyosin*	SPECT	Exposed myosin	Necrosis		85,86
^123^I-MIBG*	SPECT	Norepinephrine transporter	Sympathetic nervous system	94	94–98
^3^H-CGP12177	PET	Norepinephrine transporter	Sympathetic nervous system	99	
^13^N-Ammonia*	PET	Passive diffusion	Perfusion		110
^82^Rb-Chloride*	PET	Na^+^/K^+^-ATPase	Perfusion		105
^99m^Tc-MUGA*	SPECT	Red blood cells	Cardiac blood pool		4,102–104
^68^Ga-FAPI*	PET	Fibroblast activation	Fibrosis		116,117

*U.S. Food and Drug Administration (FDA) approved radiotracer.

References of preclinical and clinical studies examining the role of radiotracers in the context of anthracycline-induced cardiotoxicity.

## Nuclear imaging targets

### I. Metabolic dysfunction

**FIGURE 1:**
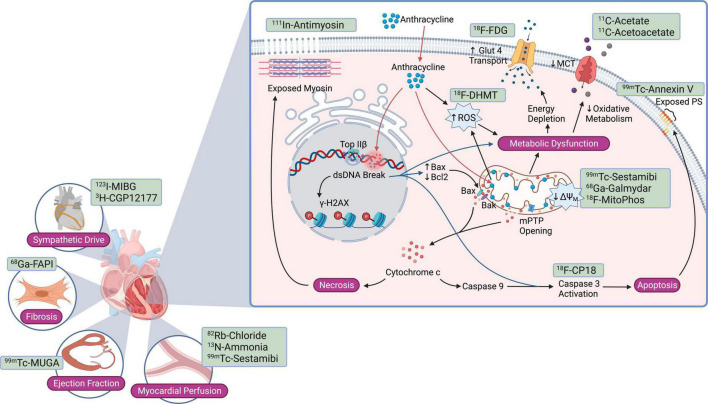
Current nuclear molecular imaging strategies targeting pathways and cell injury mechanisms activated in anthracycline-induced cardiotoxicity. Nuclear probes studied to date target anthracycline-mediated cardiomyocyte injury mechanisms such as apoptosis, necrosis, reactive oxygen species, and mitochondrial dysfunction; downstream consequences such as cardiac fibrosis; and more global changes such as sympathetic drive, myocardial blood flow, and left ventricular ejection fraction. Bax, Bcl2 associated X protein; Bak, Bcl2 antagonist/killer; Bcl2, B-cell CLL/lymphoma 2; dsDNA, double-stranded DNA; GLUT1, glucose transporter 1; γ-H2AX, phosphorylated histone variant H2AX; MCT, monocarboxylate transporter; ΔΨ_*M*_, mitochondrial membrane potential; mPTP, mitochondrial permeability transition pore; PS, phosphatidylserine; ROS, reactive oxygen species; Top IIβ, topoisomerase IIβ.

Anthracyclines induce intracellular ROS through several mechanisms. Fe^3+^ can react with the ketone and hydroxy groups of anthracyclines to form free radicals through the Fenton reaction (15). Anthracyclines also accumulate within the mitochondrial inner membrane, in part due to their high affinity to cardiolipin. In mitochondria, quinone and semiquinone moieties of anthracycline undergo redox cycling, generating large amounts of ROS (16). These events cause oxidative damage to cellular proteins, lipids, and mitochondria, resulting in mitochondrial membrane potential loss, mitochondrial swelling, activation of the mitochondrial-permeability transition pore (mPTP), and the release of cytochrome c (17). Formation of the apoptosome, initiated by cytochrome c release from mitochondria to the cytosol, leads to the cleavage and activation of caspase 3 and cell death (Figure 1). Long-term mitochondrial dysfunction also leads to a compensatory shift in cardiomyocyte metabolism, which may be targeted for AIC imaging (18).

### Glucose uptake

The most studied PET tracer for AIC imaging is ^18^F-FDG. Anthracyclines impair mitochondrial phosphorylation and the oxidation of all substrates – including fatty acids, carbohydrates, and ketones – thus driving cardiac myocytes to shift toward utilization of substrates with a more favorable ATP production phosphate/oxygen ratio, such as glucose (18, 19). Cardiomyocyte uptake of the glucose analog ^18^F-FDG is mediated by glucose transporter (GLUT) −4 and −1 (20). Whereas GLUT-1 is considered responsible for basal intracellular glucose transport, GLUT-4 and to a lesser extent GLUT-1 translocate to the plasma membrane and increase intracellular glucose uptake in response to stimuli such as insulin (21), ischemia (22, 23), anoxia (24), and catecholamines (20). Upon cardiomyocyte uptake, ^18^F-FDG is phosphorylated by hexokinase and not metabolized further (25). Additionally, the reverse reaction (dephosphorylation by glucose-6-phosphatase) is minimal (25). Thus, phosphorylated ^18^F-FDG remains trapped within cardiomyocytes. To infer glucose metabolic rate from ^18^F-FDG metabolic rate, a “lumped constant” – initially formulated by Sokoloff et al. (26) – or correction factor, is used. The lumped constant is based on competitive substrate kinetics between glucose and ^18^F-FDG, and accounts for differences in transport and phosphorylation rates (27). Importantly, the lumped constant for ^18^F-FDG in the myocardium is dependent on fasting state and serum insulin levels (28–31). The lumped constant has not been evaluated in the setting of anthracycline chemotherapy-induced cardiac injury.

Preclinical studies have assessed the potential utility of ^18^F-FDG in AIC. Bulten et al. observed a progressive increase in ^18^F-FDG uptake in mice treated with doxorubicin (15 mg/kg, once every 3 weeks for up to four cycles) (32). They further noted a significant correlation between myocardial ^18^F-FDG uptake and hypoxia-inducible factor (HIF)-1α, a hypoxia-driven transcription factor that activates GLUTs and glycolytic enzymes. In another study of doxorubicin treated mice, increased ^18^F-FDG myocardial uptake had a direct correlation with histologically determined myocardial redox stress (33).

Several retrospective clinical studies have documented higher ^18^F-FDG uptake in patients treated with anthracyclines. Borde et al. observed in lymphoma patients treated with doxorubicin-based chemotherapy a higher post-therapy ^18^F-FDG uptake than before treatment (34). In a retrospective study of 43 Hodgkin lymphoma patients that developed AIC, Sarocchi et al. observed that a decrease in LVEF several months to years following treatment was inversely correlated with LV uptake of ^18^F-FDG during doxorubicin containing chemotherapeutic treatment (R^2^ = 0.30, *P* < 0.01) (35). Similarly, in a recent study of 121 consecutive breast cancer patients undergoing treatment with anthracycline or trastuzumab, Kim et al. found that patients who developed cardiotoxicity had a higher ^18^F-FDG right ventricular uptake than patients who did not (2.4 ± 1.1 vs. 1.6 ± 0.7, *P* = 0.012) (36). Though it remains unclear whether the observed right ventricular uptake preceded or was a result of LV dysfunction, other have reported that left ventricular and right ventricular global longitudinal strain are both similarly impaired during trastuzumab treatment (37). Additional studies have suggested that baseline LV ^18^F-FDG SUV may also be an indicator of patient susceptibility to AIC. Bauckneht et al. observed that among a cohort of 36 Hodgkin lymphoma patients that had previously undergone doxorubicin treatment, the 11 patients that developed significant post treatment reduction in ejection fraction had lower pre-treatment LV ^18^F-FDG uptake compared to the remaining 25 patients that didn’t develop cardiac abnormalities (mean SUV = 1.53 ± 0.9 vs. 3.34 ± 2.54, *P* < 0.01) (38). Heckmann et al. (39) demonstrated in a retrospective study (*n* = 337 consecutive patients) that Hodgkin’s lymphoma (*n* = 52) was associated with a higher cardiac ^18^F-FDG uptake (mean SUV = 3.5 ± 3.6, odds ratio = 2.4, *P* < 0.01) whereas non-Hodgkin’s lymphoma (*n* = 57) and non-lymphatic cancer (*n* = 228) were not. Interestingly, the authors observed that the increase in cardiac ^18^F-FDG uptake in Hodgkin’s lymphoma was not determined by prior chemotherapy and/or serum glucose levels, however, with the caveat that patient preparations were not optimized or standardized for cardiac ^18^F-FDG PET imaging, in addition to the retrospective nature of the study (39). A significant limitation of these retrospective studies is that protocols were designed for cancer staging and not to measure cardiomyocyte ^18^F-FDG uptake *per se*, which were done *post hoc* in a retrospective manner. In this setting, patients were only required to fast a minimum of 6 h during which myocardial metabolic patterns still have a high degree of variability (40). Furthermore, the pattern of myocardial ^18^F-FDG uptake may also need to be taken into consideration when determining physiologic vs. pathologic signals (41). Whereas standardized protocols incorporating adequate nutritional preparation are required (42), these preliminary studies are promising and set the stage for the prospective evaluation of cardiomyocyte ^18^F-FDG uptake in AIC.

### Oxidative metabolism

Acetate is utilized by cardiomyocytes in the tricarboxylic acid (TCA) cycle and can thus serve as a metric to quantify myocardial oxygen consumption. A preclinical model of chronic doxorubicin treatment in rats (2 mg/kg IV weekly for 6 weeks) observed that doxorubicin decreased myocardial oxygen consumption reserve (2.3 ± 0.3 vs. 1.8 ± 0.4, *P* = 0.02) (43). ^11^C- acetoacetate, a ketone body that utilizes the same monocarboxylic acid transporter as acetate, exhibited similar changes in an analogous rat model treated with doxorubicin (44).

Nony et al. investigated ^11^C-acetate uptake to assess myocardial oxidative metabolism and myocardial blood flow in patients treated with anthracyclines (45). The resting myocardial blood flow of 6 patients were serially measured during a doxorubicin treatment course of 50 mg/m^2^ every 3 weeks for 15 weeks (cumulative dose of 300 mg/m^2^). The investigators observed that compared to baseline, there was no significant change in resting myocardial blood flow during or after completion of doxorubicin treatment. Similarly, no significant changes were noted in K_*mono*_, an index of myocardial oxygen consumption (45, 46).

### Fatty acid metabolism

Whereas 70 to 90% of cardiac ATP production is derived from fatty acid β-oxidation under physiologic conditions, fatty acid usage decreases significantly in heart failure and cardiomyopathy models (47), thus making it a possible target for AIC imaging. There are no preclinical or clinical studies to date that have applied radiolabeled fatty acids to monitor AIC. Potential candidates include ^11^C-palmitate, although its clinical utility is hindered by a lack of kinetic data that models and accounts for the redistribution of ^11^C metabolites within various lipid pools (48). ^18^F-FTHA (14[R,S]-^18^F-fluoro-6-thia-heptadecanoic acid) is another PET tracer that could circumvent this limitation by utilizing a sulfur atom in its backbone that prevents it from undergoing further β-oxidation (49).

### Mitochondrial membrane potential

^99m^Tc-sestamibi is a SPECT tracer clinically used to image myocardial perfusion, though its utility as a lipophilic cation has proven useful to detect disruptions in mitochondrial membrane potential. The ability of these cations to accumulate inside the mitochondria has been used as a proxy index for mitochondrial membrane potential (ΔΨ_*M*_) (50). Animal studies conducted by Safee et al. indicated that rats treated with a single dose of doxorubicin had lower levels of ^99*m*^Tc-sestamibi uptake in the myocardium, corresponding to a loss in ΔΨ_*M*_. A significant 2.5-fold decrease in ^99*m*^Tc-sestamibi 2 weeks post-treatment was detected only in rats treated with the highest doxorubicin dose (10 mg/kg), which was associated with a 7% and 9.5% drop in ejection fraction and fractional shortening, respectively, that was also only significant 2 weeks post-treatment (51). While promising, usage of ^99*m*^Tc-sestamibi is hindered by its pharmacokinetics and the limited sensitivity inherent to most SPECT tracers. In comparison, PET tracers such as the metalloprobe ^68^Ga-galmydar have been developed for this same application (52). In live-cell fluorescent imaging of H9c2 cells, Sivapackiam et al. observed a dose- and time-dependent decrease in ^68^Ga-galmydar that correlated with an increase of caspase-3 activation (52). These findings were subsequently confirmed in *in vivo* models, where the authors reported a nearly 2-fold decrease in myocardial ^68^Ga-galmydar uptake 5 days following a single doxorubicin dose of 15 mg/kg in rats, verified by post-imaging quantitative biodistribution. Another PET lipophilic cation that bears promise is [1-(2-^18^F-fluoroethyl),1H[1,2,3]triazole-4-ethylene]triphenylphosphonium bromide (^18^F-MitoPhos) (53). In a Langendorff perfusion heart model, ^18^F-MitoPhos exhibited more than double cardiac retention compared to ^99*m*^Tc-sestamibi. Moreover, *in vivo* studies in an acute doxorubicin rat model indicated a close to 50% decrease in the left ventricular retention of ^18^F-MitoPhos compared to controls 48 h after treatment. While ^68^Ga-galmydar and ^18^F-MitoPhos represent encouraging alternatives to ^99*m*^Tc-sestamibi for measuring ΔΨ_*M*_, further studies are needed to corroborate these findings with reference parameters such as ejection fraction and fractional shortening.

An additional promising strategy for the scrutiny of ΔΨ_M_ involves the radiolabeled lipophilic cation ^18^F-tetraphenylphosphonium (^18^F-TPP^+^) (54). Using a pig model, Alpert et al. demonstrated its *in vivo* myocardial applicability *via* a novel method accounting for extracellular space and employing kinetic analysis to estimate tracer volume of distribution (55). Additionally, Pelletier-Galarneau et al. demonstrated excellent agreement of *in vivo* measures of myocardial ^18^F-TPP^+^ in healthy humans subjects with previous *in vitro* assessments (56), paving the way for human studies quantifying temporal changes in mitochondrial membrane potential using this radiopharmaceutical in the context of anthracycline-induced cardiotoxicity (57). These tracers require prospective clinical trial evaluation to determine their clinical utility.

### Reactive oxygen species

[^18^F]6-{4-[(1-(2-fluoroethyl)-1*H*-1,2,3-triazol-4-yl) methoxy]phenyl}-5-methyl-5,6-dihydrophenanthridine-3,8- diamine (^18^F-DHMT), an analog of the superoxide indicator dihydroethidium, has previously been identified as a promising PET radiotracer of ROS generation (58). In a chronic doxorubicin-induced cardiotoxicity rodent model, Boutagy et al. described the significant increase in myocardial ^18^F-DHMT uptake 4 weeks post treatment which preceded degradation in LVEF that was significant only 6 weeks post treatment (*P* = 0.0012) (59). Correlation analysis suggested an inverse correlation (r^2^ = 0.6; *P* = 0.01) between LV ^18^F-DHMT uptake and LVEF as well as a direct correlation (r^2^ = 0.72; *P* = 0.007) between LV ^18^F-DMHT and LV ESV. These results suggest that ^18^F-DHMT may be a viable radiotracer for early assessment of cardiotoxicity that precedes left ventricular systolic dysfunction.

### II. Cell death

Dox intercalates in the DNA and induces single- and double-strand DNA breaks in target cells in a topoisomerase (Top)-2-dependent manner (60). By producing temporary single- or double-stranded DNA breaks, Top regulates topological changes during DNA replication, transcription, or recombination (61). Top-2α is overexpressed in tumors and is the molecular basis of Dox anticancer activity (62, 63).

Adult cardiomyocytes express Top-2β but not Top-2α (62), and Top-2β is also a Dox target, forming a Top-2β-Dox-DNA ternary cleavage complex that induces DNA strand breaks and ensuing cell death (64, 65). These DNA breaks rapidly result in the phosphorylation of histone variant γ-H2AX, a sensitive marker of the DNA damage response (66, 67) (Figure 1). Subsequently, mediator of DNA damage checkpoint protein (MDC)-1 binds to γ-H2AX (68) and facilitates DNA damage repair protein recruitment (69, 70). Furthermore, Dox/Top-2β bind to selective promoters, significantly affecting the cardiomyocyte transcriptome (65, 71). Ensuingly, key antioxidative enzymes are reduced, providing a mechanism linking Dox-induced reactive oxygen species (ROS) production in a Top-2β-dependent manner. For example, peroxisome proliferator activated receptor-γ (PPAR-γ) coactivator –1-α and -β, pivotal transcription factors implicated in mitochondrial biogenesis, are decreased in the setting of Dox cardiotoxicity (65, 71).

**FIGURE 2:**
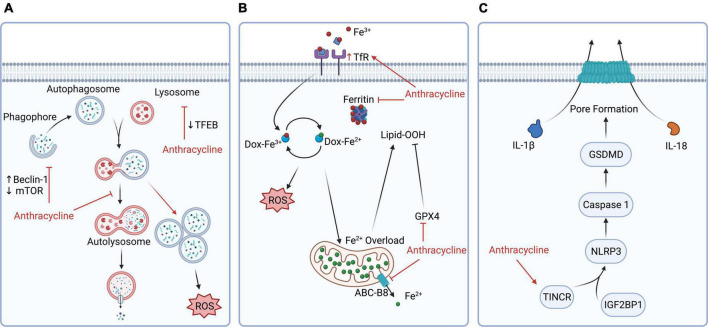
Additional anthracycline cardiotoxicity mechanisms for the development of novel molecular nuclear probes. Highlighted mechanisms have not yet been targeted using a nuclear imaging strategy, and certain key molecular or biochemical targets are presented for the potential development of new SPECT/PET tracers. **(A)** Anthracyclines impair autophagy by compromising lysosomal acidification, increasing beclin-1 expression, inhibiting mTOR, and inhibiting transcription factor EB, thereby blocking autophagic flux and causing an accumulation of autolysosomes that leads to increased ROS production. New probes may be developed to target molecules implicated in the accumulation of undegraded autolysosomes. **(B)** Anthracycline-induced ferroptosis is caused by iron overload through upregulation of TfR, inactivation of ferritin, inhibition of ABC-B8, and downregulation of GPX4, which may be targets for probe development. **(C)** Anthracycline induces pyroptosis by upregulating TINCR, which increases the expression of NLRP3 and caspase 1 activation. Future probes may for example target NLRP3 or caspase1. ABC-B8, ATP binding cassette subfamily B member 8; Dox, doxorubicin; GPX4, glutathione peroxidase 4; GSDMD, gasdermin D; IGF2BP1, insulin-like growth factor 2 mRNA binding protein 1; IL, interleukin; Lipid-OOH, lipid hydroperoxides; mTOR, mammalian target of rapamycin; NLRP3, nucleotide-binding oligomerization domain (NOD-), leucine-rich repeat (LRR-), and pyrin domain (PYD)-containing protein 3; ROS, reactive oxygen species; TFEB, transcription factor EB; TfR, transferrin receptor; TINCR, terminal differentiation-induced non-coding RNA.

Anthracyclines triggers various cell death mechanisms (Figures 1, 2), though the two most well characterized pathways in AIC are apoptosis and necrosis, mediated in part by Bax-induced mitochondrial damage (Figure 1). Bax (Bcl-2 associated X protein) is a member of the Bcl-2 family. Under homeostatic conditions, Bax resides in an inactive conformation in the cytosol (72). Upon anthracycline treatment, Bax undergoes a conformational change that results in its translocation to the mitochondrial membrane (Figure 1). There, Bax mediates opening of the mitochondrial permeability transition pore (mPTP) located in the inner mitochondrial membrane (73). In turn, mPTP opening leads to swelling of the mitochondrial intermembrane space followed by rupture of the outer mitochondrial membrane, release of intermembrane space proteins – including the small soluble electron carrier cytochrome c – into the cytosol, and cardiomyocyte necrosis (74) (Figure 1). Another proposed mechanism of anthracycline-induced, Bax-mediated cytochrome c release is the oligomerization of Bak and Bax within the outer mitochondrial membrane, leading to its permeabilization and activation of apoptotic pathways (72, 75).

### Annexin V

Annexin V is a well-established method of detecting externalized phosphatidylserine (PS), a phospholipid that is translocated from the inner to the outer leaflet of the plasma membrane early in apoptosis. ^99*m*^Tc-annexin-V was scrutinized by Bennink et al. in an acute doxorubicin-induced cardiotoxicity rat model. Doxorubicin treated rats displayed a significant increase in myocardial ^99*m*^Tc-annexin-V uptake, with longer doxorubicin treatment regimens corresponding to an even higher uptake (76). Furthermore, heart-to-body weight ratio decreased in response to doxorubicin treatment, which may have been an indication of cardiomyocyte death. These findings correlated well with cardiotoxicity measured through immunohistochemistry and the TUNEL assay. Clinical studies utilizing annexin V probes have been limited; ^99*m*^Tc-HYNIC-annexin-V, was tested in early clinical trials but its performance in detecting apoptosis in head and neck carcinoma was limited by moderate non-specific binding and slow clearance times (77).

### Caspase

A more direct way of measuring apoptosis is by targeting caspase activation. Caspases are intracellular enzymes that are essential for executing apoptosis. Different initiator caspases can be activated extrinsically or intrinsically, though both pathways ultimately converge with caspase-3 and –7 activation. Several analogs of 5-Dialkylaminosulfonylisatins, a potent non-peptide inhibitor of caspase-3 and –7, have been adapted as PET tracers. ^18^F-ICMT-11, ^11^C-WC-98, and ^18^F-WC-IV-3 have demonstrated high caspase-3 affinity *in vitro* but all exhibited poor specificity *in vivo* models, likely due to the dicarbonyl moiety caspase binding region of isatin being recognized by other proteases such as cathepsins (78–80). A substrate-based probe, ^18^F-CP18, was designed to improve specificity by taking advantage of caspase-3′s unique substrate recognition motif for aspartic acid residues in the P1 and P4 positions (75 - 81). In this probe, the caspase-3 substrate sequence Asp-Glu-Val-Asp connects a radioactively labeled metabolite to a short polyethylene glycol (PEG) chain. The hydrophilic PEG chain facilitates the probe’s transport across the cell membrane and upon encountering activated caspase-3 it is cleaved away, leaving the radioactively labeled metabolite inside the cell. While initially developed for visualization of apoptosis in tumors, this radiotracer has also been adapted for AIC imaging (82). Su et al. (83) detected increased accumulation of ^18^F-CP18 in the myocardium starting at 3 weeks after doxorubicin treatment in mice, which was histologically validated using a TUNEL assay. The authors also observed that ^18^F-CP18 detects myocardial apoptosis at a stage prior to significant changes in LVEF. Clinical studies are required to further expand on these promising results.

### Necrosis

^111^In-antimyosin is a tracer that binds to the exposed myosin of damaged cells, an indicator used to quantify regions of myocardial necrosis. Early clinical studies evaluated ^111^In-antimyosin in AIC (84–86). Carrió et al. studied 30 sarcoma patients who underwent serial ^111^In-antimyosin imaging prior to chemotherapy and at intermediate (240–300 mg/m^2)^ and maximal (420–600 mg/m^2^) cumulative doxorubicin doses. Whereas an abnormal heart-to-lung ^111^In-antimyosin uptake ratio was observed with both doxorubicin dosages, maximal exposure to doxorubicin led to a more pronounced ^111^In-antimyosin uptake with a ratio of 2.02 ± 0.3 (*P* < 0.01) and was associated with a significant ≥10% decrease in LVEF (85). Furthermore, in a follow up study of patients that had discontinued anthracycline treatment due to a decrease in LV function leading to a LVEF <50%, Olmos et al. demonstrated that patients with an ^111^In-antimyosin heart-to-lung uptake ratio ≥1.87 experienced a persistent decline in LVEF at 2–26 weeks follow-up, with 4 out of 11 of these patients subsequently developing congestive heart failure (86). In contrast, patients with a transient change in LVEF following the discontinuation of anthracycline treatment had a mean ^111^In-antimyosin heart-to-lung uptake ratio of 1.52. Despite these promising results, interest in ^111^In-antimyosin cardiac imaging has waned and this tracer is not commonly used in contemporary practice due to its detection of necrotic cell death which occurs at more at more advanced stages of the disease process and thereby limits options for clinical intervention.

### III. Sympathetic innervation

The clinical signs of early myocardial cell injury are often masked by a compensatory rise in sympathetic drive. Indeed, an increase in chronotropy and inotropy preserve LVEF during the early stages of AIC (87, 88). While initially beneficial, long term cardiac sympathetic activation is detrimental, with cardiac sympathetic dysinnervation occurring in cardiomyopathy and heart failure (89). The main catecholamine released by sympathetic postganglionic fibers is norepinephrine which accounts for 70% of circulating levels, with the remainder mainly released by the adrenal gland (90, 91). The majority of clinical tracers that monitor sympathetic innervation are radiolabeled analogs of norepinephrine.

### ^123^I-metaiodobenzylguanidine

^123^I-metaiodobenzylguanidine (^123^I-MIBG), a norepinephrine analog that shares with it similar release and uptake mechanisms, can be used to identify areas of abnormal adrenergic innervation in the myocardium. Approximately 80–90% of norepinephrine released at sympathetic nerve terminals is taken up again *via* norepinephrine transporter uptake-1 (89). Reduction of norepinephrine uptake at these sites has been documented in various cardiovascular diseases (92, 93). Like norepinephrine, reuptake of ^123^I-MIBG is mediated by norepinephrine transporters along myocardial sympathetic nerve terminals. Given ^123^I-MIBG is not metabolized, its retention can be used as an indicator for neuronal integrity.

^123^I-metaiodobenzylguanidine uptake is reduced in preclinical models of AIC. In a chronic doxorubicin rat model (2 mg/kg IV weekly for 1, 2, 3, 4, 5 and 8 weeks), a significant decrease in cardiac ^123^I-MIBG uptake was detected at week 4, which correlated with histologically examined myocardial tissue damage (94). A comparative study of ^123^I-MIBG with ^18^F-FDG in a chronic doxorubicin-induced cardiotoxicity rat model (15 mg/kg cumulative dose) indicated that ^18^F-FDG uptake decreased significantly in doxorubicin treated groups at weeks 4 and 6 (4.2 ± 0.5%ID/g vs. 9.2 ± 0.8%ID/g at week 6), which correlated with LVEF (*r* = 0.49, *P* = 0.002) (95). In contrast, a significant decrease in ^123^I-MIBG heart-to-mediastinum (H/M) ratio between groups was detected earlier at week 2 (∼1.9% vs. ∼1.4%, *P* < 0.05), maintained at weeks 4 and 6, but was not correlated with LVEF decrease at week 6 (*r* = 0.24, *P* = 0.15). While promising, a limiting point of these preclinical studies is the large variability in anthracycline doses and temporal administration patterns which may lead to systemic toxicity in animals.

Early and late H/M ratios of ^123^I-MIBG uptake were proposed as an index for stratifying prognosis and risk in patients with chronic heart failure (96). Carrió et al. (96) observed a significant 1.5-fold decrease in cardiac ^123^I-MIBG uptake in sarcoma patients undergoing maximal cumulative doxorubicin treatment (420–600 mg/m^2^), which also corresponded with a significant (≥10%) reduction in LVEF. A similar reduction in ejection fraction was not detected in patients at intermediate cumulative doxorubicin doses (240–300 mg/m^2^), though 25% of patients exhibited slight decreases in ^123^I-MIBG uptake that were not significant compared to baseline. A study by Laursen et al. (97) further reinforced that ^123^I-MIBG imaging is only applicable in patients undergoing high cumulative doxorubicin doses. Moreover, the authors observed that patients undergoing intermediate cumulative doses of doxorubicin had a non-significant increase post therapy on both background-corrected WOR – which reflects norepinephrine retention in adrenergic neurons, closely connected to sympathetic tone (18.6% vs. 23.4%, *P* = 0.09), and H/M_*early*_ – a measure of the anatomical distribution of functioning myocardial adrenergic neurons (2.7% vs. 2.9%, *P* = 0.4). No association was observed between follow-up decreases in LVEF and WOR (*P* = 0.5). Moreover, in a study of asymptomatic patients who had completed anthracycline treatment ≥2 years prior, ^123^I-MIBG uptake did not differ between anthracycline treated patients (cumulative anthracycline dose 257.6 ± 117.1 g/m^2^) and control patients, as assessed either by mean H/M_*late*_ – which reflects overall neuronal functioning, i.e., the product of norepinephrine uptake, storage, and release (2.24% vs. 2.26%, *P* = 0.5) – or WOR (10.32% vs. 9.64%, *P* = 0.8) (98). These results suggest that the myocardial adrenergic activation initially detected by ^123^I-MIBG during treatment is reversible upon discontinuation of anthracycline treatment. Overall, ^123^I-MIBG adrenergic imaging is not presently a clinically applicable strategy for the early detection of AIC, though further clinical studies may be warranted to investigate its sensitivity at lower cumulative anthracycline doses.

### Additional tracers

Other PET radiotracers for cardiac sympathetic innervation have been studied in animal models. *Ex vivo* biodistribution studies 3 weeks after chronic doxorubicin treatment in rats found no change in cardiac uptake of the norepinephrine analog ^11^C-*meta*-hydroxyephedrine (^11^C-HED) or the phosphodiesterase-4 inhibitor (*R*)-^11^C-rolipram (which provides an index of myocardial cyclic AMP activity, downstream of norepinephrine) (99). On the other hand, the authors noted a decreased uptake of the β-adrenergic antagonist ^3^H-CGP12177 3 weeks post treatment while there was no change in ejection fraction or heart-to-weight ratio. Similarly, Kizaki et al. also reported that β-adrenergic receptor gene expression decreases following doxorubicin treatment in rats (100).

### IV. Myocardial function, perfusion, and blood flow

^99m^Tc-MUGA imaging is an accurate method to assess ventricular contraction. While generally avoided in pediatric patients due to radiation concerns, ^99*m*^Tc-MUGA may be used to monitor cardiotoxicity in adult patients due to its high accuracy and low inter-observer variability (101–103). In a prospective study of 28 non-Hodgkin lymphoma patients undergoing doxorubicin treatment, Nousiainen et al. observed that a decrease of ≥4% in LVEF quantified by ^99*m*^Tc-MUGA following a cumulative doxorubicin dose of 200 mg/m^2^ predicted AIC with 90% sensitivity and 72% specificity (104). However, these findings were not supported by a large-scale retrospective study of 630 patients grouped according to increasing doxorubicin doses or placebo (4). Overall, assessment of LVEF – even if done accurately by ^99*m*^Tc-MUGA – is not a favored approach to assess AIC as it only provides a global and often delayed assessment of cardiac mechanical abnormalities following injury.

Microvascular dysfunction is a possible complication of AIC (105). The SPECT myocardial perfusion imaging tracer ^99*m*^Tc-sestamibi was assessed in AIC in a prospective study of breast cancer patients undergoing radiation therapy and doxorubicin treatment (106). Hardenbergh et al. observed that 7 out of 10 patients developed new visible perfusion defects 6 months post-radiation. More recently, PET tracers such as ^13^N-ammonia and ^82^Rb-chloride have become more common for myocardial perfusion assessment due to their superior diagnostic accuracy compared to SPECT (107–109). In a small prospective study (*n* = 10) using ^13^N-ammonia, Nehmeh et al. reported decreased MFR 1-year post-radiation in 50% of breast cancer patients (*n* = 4) receiving radiotherapy and anthracycline (110). However, these findings are suggested by a small dataset only, and it is unknown if the observed cardiotoxic effects were caused by the anthracycline treatment or the radiation. PET myocardial perfusion tracers are amenable to myocardial blood flow quantitation, an integrated measure of epicardial and microvascular coronary artery disease (109). ^82^Rb-chloride assessment of myocardial blood flow in lymphoma patients by Laursen et al. revealed a mild reduction in myocardial flow reserve (MFR: 2.69 vs. 2.51, *P* = 0.03) – calculated as the stress/rest ratio of myocardial blood flow – 3 days after initial doxorubicin treatment, with 13 out of 54 patients exhibiting low cardiotoxicity threshold (>20% decline in MFR) (105). Importantly, the MFR decline was independent of perfusion defects determined using the summed stress score and summed difference score. Stratifying patient risk of developing AIC in this manner is relevantly new, and additional studies are needed to determine whether an acute reduction in MFR shortly after anthracycline administration may identify patients at higher cardiotoxicity risk. Furthermore, the PET tracers ^13^N-ammonia (110), ^15^O-water (111) and ^18^F-flurpiridaz (112, 113) should also be considered to assess myocardial blood flow changes in AIC given their higher myocardial extraction fractions (108).

### V. Cardiac fibrosis

Myocardial fibrosis (114) may occur as a result of AIC. While the exact role cardiac fibroblasts play in AIC remains underexplored, doxorubicin was recently reported to induce fibroblast differentiation (115). ^68^Ga-fibroblast activation protein inhibitor (FAPI) is a robust fibroblast PET tracer that was initially developed to detect high FAP-expressing, cancer-associated fibroblasts. In a retrospective study of *n* = 32 patients, Siebermair et al. evaluated the cardiac uptake of ^68^Ga-FAPI in a heterogenous population of cancer patients treated with various anticancer therapies (116). While only 3 patients with FAPI uptake had been treated with anthracyclines, the authors observed a significant association of myocardial FAPI uptake with CAD and LVEF. Using modeling (*n* = 185) and confirmatory (*n* = 44) consecutive cohorts of patients with cancer metastasis who had FAPI-positive PET scans, Heckmann et al. (117) did not observe an association of focal myocardial FAPI uptake with anthracycline treatment, however, noted a correlation of high signal intensities with cardiovascular risk factors and metabolic disease. Additional studies are warranted to investigate cardiac fibroblast FAP expression patterns in the context of AIC (116).

## Additional anthracycline cardiotoxicity mechanisms for future development of candidate molecular targets

Several AIC mechanisms have not been targeted to date using a nuclear imaging strategy. Anthracycline-induced cardiac injury is multi-factorial and -genic, with no single mechanism fully explaining all aspects of the injury process. Importantly, anthracyclines induce all forms of cell death (118, 119). We highlight below three additional injury pathways that may lead to the development of new radiotracers for nuclear molecular imaging in AIC.

### Autophagy

Autophagy is a homeostatic process in which cells utilize lysosomes to remove unnecessary or damaged cellular components (75). Anthracyclines block cardiomyocyte autophagic flux by impairing lysosomal acidification – critical for lysosomal hydrolytic enzyme activity and lysosomal maturation (120) – leading to the accumulation of undegraded autolysosomes (121) (Figure 2A). Another mechanism of anthracycline interference with autophagic flux is the inhibition of transcription factor EB expression which is a regulator of lysosomal proteolysis mediated primarily by cathepsin B activity (122). Furthermore, phosphoinositide 3-kinase γ (PI3Kγ) is induced downstream of Toll-like receptor 9 by cardiomyocytes following anthracycline treatment (123). PI3Kγ leads to Akt phosphorylation and inactivation of mTOR (mammalian target of rapamycin) targets, thus causing autophagy inhibition and a reduced ability to remove damaged organelles such as mitochondria (123). Expression of beclin-1, a mediator of autophagy initiation, increases following doxorubicin treatment in mice (121). Furthermore, Li et al. indicated that haploinsufficiency of beclin-1 diminishes autophagy initiation, leading to fewer unprocessed autolysosomes and decreased ROS production. Conversely, doxorubicin-induced cardiac injury is accentuated in mice with beclin-1 overexpression. Taken together, these mechanisms indicate the contribution of autophagy perturbation to AIC-induced cardiomyocyte death, cardiac remodeling, and failure (124).

### Ferroptosis

Ferroptosis is a type of cell death characterized by the iron-related accumulation of lipid peroxides (75). Iron plays a significant role in AIC injury (125) (Figure 2B). Heart biopsies of patients who experienced anthracycline-related heart failure demonstrated excessive mitochondrial iron accumulation (126). This excessive iron load in the mitochondria can be explained by doxorubicin downregulation of ATP-binding cassette protein-B8 (ABC-B8), which mitigates iron transport out of the mitochondria (126). Doxorubicin also downregulates the key anti-ferroptosis protein glutathione peroxidase-4, resulting in lipid peroxidation. Additionally, doxorubicin can interact with the iron response elements of ferritin, reducing cytosolic ferritin and increasing labile iron (127).

### Pyroptosis

Pyroptosis is a cell death mechanism characterized by increased proinflammatory signaling and activation of caspase-1, –4, –5, and –11, leading to plasma membrane rupture mediated by gasdermin D (GSDMD) (75). Previous research has determined that doxorubicin induces pyroptosis *via* induction of terminal differentiation-induced non-coding RNA (TINCR) and activation of the NLRP-3-caspase-1 pathway (128–130). Specifically, doxorubicin upregulation of TINCR leads to recruitment of the adapter protein IGF2BP1 (insulin-like growth factor 2 mRNA-binding protein 1) and stabilization of NLRP3 mRNA (128). This increases NLRP3 expression and activates caspase-1, thereby leading to GSDMD cleavage, plasma membrane rupture, and interleukin (IL)-1β and IL-18 release (Figure 2C).

## Need for improved cardiac imaging approaches in the context of contemporary anthracycline use

While cancer affects more than one in three people over their lifetime, improved long-term cancer survival has led to an increase in the incidence of adverse cardiac side-effects of cancer treatments (131). The U.S. National Cancer Institute estimates that in 2022 there will be ∼ 18 million cancer survivors which mounts to >5% of the U.S. population (132). Anthracyclines are a cornerstone of chemotherapy in various cancers (133), however, their use is complicated by anthracycline-induced cardiotoxicity (134, 135) which has been appreciated for decades (4, 136–138).

Despite the continued discovery of alternative chemotherapeutic strategies, and their known cardiotoxic side-effects, anthracyclines remain a mainstay of many cancer treatments (139, 140). Indeed, anthracyclines are used in 30–35% of breast cancer patients (141–143) and 60–70% of elderly lymphoma patients (144, 145). Moreover, 50–60% of childhood cancer survivors were treated with a chemotherapy regimen containing anthracyclines (146, 147). In parallel, continued advances in cancer therapy have increased the survival rate of childhood cancer to ∼ 80% (132). Furthermore, long-term follow-up of childhood cancer survivors indicate that up to 30% of patients treated with anthracyclines have signs of cardiac dysfunction in adulthood that are unmasked when more sensitive detection techniques are used (148), indicating significant under-estimation of long-term complications. These observations highlight the need for additional and improved imaging strategies in the context of AIC.

While echocardiography continues to be the most widely used tool for AIC monitoring, it is important to consider the limitations and advantages of each modality when selecting the proper screening exam for an individual patient. Echocardiography enjoys attractive features such as wide availability, rapid interpretation, lack of ionizing radiation exposure and relatively low cost which have firmly established it as the staple of AIC monitoring (6). However, the quality of the study is highly dependent on patient anatomy, acoustic windows, technician skill, and interobserver variability to a greater degree than other available methods (149–151).

In contrast, automated segmentation and ventricular volume algorithms offer precise and accurate evaluations of chamber function in cardiac MRI and multi-detector gated cardiac CT. However, cardiac MRI is more expensive and less widely available at many clinical centers. It is also a time-consuming exam that requires significant patient cooperation and can be undermined by rapid heart rates, arrhythmias, or by the presence of intrathoracic hardware such as implantable pacemakers or defibrillators that can introduce excessive signal artifact (152, 153).

Cardiac CT is also challenged by susceptibility to gating artifacts with rapid or irregular heart rhythms, need for iodinated contrast which is limiting in patients with kidney disease, as well as less robust data on the assessment of muscle strain or mechanics that can indicate early toxicity as compared to echocardiography or cardiac MRI (154).

Lastly, although largely fallen in clinical desuetude, patients with poor echocardiographic acoustic windows and contraindications to MRI or CT imaging may be referred for a radionuclide MUGA scan. This modality has proven to be a viable alternative to cardiac MRI or CT in patients where the precision or accuracy of echocardiographic measurements are in question, and thus remains a clinical option when choosing a strategy to monitor for AIC (155, 156). However, the advent of unique and specific markers in the growing field of nuclear molecular imaging may offer an additional toolset to detect and treat early manifestations of AIC.

Whereas the utility of perfusion-based imaging and myocardial blood flow quantitation for the detection of sub-clinical or early AIC has been studied in several small trials, additional research is needed to bring the evidence to the level of routine clinical utility. In particular, PET-derived myocardial blood flow has been examined as a potential marker of patients who may be at increased risk for AIC (157, 158). Whether changes in myocardial blood flow metrics are a sign of early cardiac stress or indicative of irreversible toxicity in response to anthracyclines require further scrutiny (157). If the value of myocardial flow reserve assessment in AIC can be further demonstrated in large-scale clinical studies, it could be added as an early evaluation strategy in company of echo-derived left ventricular global longitudinal strain or biomarkers such as plasma troponin levels.

Similarly, although no societal guidelines exist for routine myocardial perfusion imaging of patients undergoing anthracycline based chemotherapy regimens, the high correlation of CAD and subsequent acute coronary syndromes in cancer patients often prompts clinicians to screen patients with intermediate or high risk of CAD prior to initiation of therapy. In one retrospective analysis of 6.5 million cases of acute coronary syndromes, 9% of the patients had a diagnosis of cancer, either active or in remission, suggesting that pre-chemotherapy evaluation and revascularization, if indicated, may be appropriate in this patient group (159).

Several of the probes discussed in this review such as ^18^F-FDG and ^68^Ga-FAPI are used for cancer staging or progression evaluation. Retrospective analyses of these clinical studies suggest abnormal myocardial uptake in certain patients, paving the way for dedicated cardiac studies to detect and monitor AIC. There is also an opportunity to investigate these probes for oncologic and cardiac assessment in tandem. Doing so, however, will require patients to have a standardized preparation and clinicians to follow a more rigid imaging protocol that adheres to both oncologic and cardiac quality control requirements.

**FIGURE 3:**
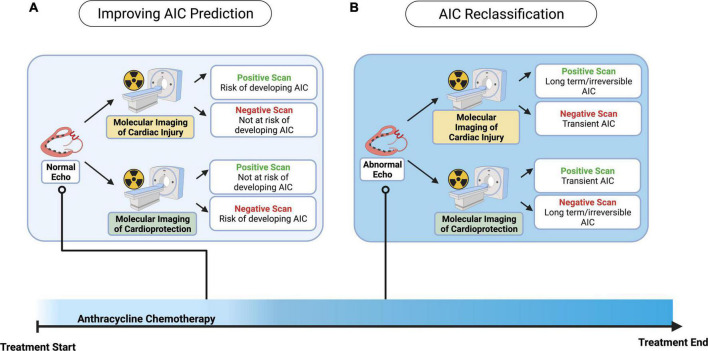
Potential clinical applications of nuclear molecular imaging in predicting and reclassifying cardiotoxicity in cancer patients undergoing anthracycline treatment. Different clinical algorithms may be followed according to the mechanistic pathway targeted for imaging – whether expression of cardiac injury related proteins, or alternatively activation of cardioprotective pathways – and the presence or absence of abnormal echocardiographic features suggestive of cardiotoxicity. **(A)** Molecular imaging may be used to predict the risk of developing cardiotoxicity during and/or following anthracycline treatment in patients with a normal echocardiogram. **(B)** In patients with an abnormal echocardiogram suggesting a new impairment in cardiac function, molecular imaging may be able to finetune the cardiotoxicity diagnosis with implications on the duration of anthracycline-induced cardiac dysfunction, or to reclassify the echocardiographic diagnosis, thereby providing oncologists and cardiologists greater diagnostic certainty with ensuing clinical implications.

Echocardiography, including the segmental assessment of myocardial function by strain or displacement vectors, remains the first imaging modality of choice for AIC screening. Therefore, we posit a complementary role – dependent on the imaging targets – for the clinical utilization of nuclear molecular imaging applications in cardio-oncology, that we separate into two aims: 1) improving the prediction of AIC development in the setting of a normal echocardiogram, and 2) improving AIC reclassification in the setting of an abnormal echocardiogram (Figure 3). The clinical adoption of nuclear molecular imaging approaches remains limited at present, however, is poised to significantly affect current imaging strategies that have limitations in both sensitivity and specificity to screen and monitor AIC.

## Conclusion

Anthracycline-induced cardiotoxicity involves a broad range of pathophysiological pathways that lead to cardiomyocyte injury and that may be further complicated by cardiomyopathy and heart failure. Mechanisms implicated in the disease process, as well as molecular responses thereto, can be probed for nuclear imaging, e.g., metabolic dysfunction, cardiomyocyte death, sympathetic innervation, and changes in myocardial blood flow. Detecting these AIC-induced processes at a subclinical level, prior to the onset of irreversible cardiac impairment, may provide clinicians with valuable information permitting changes in chemotherapeutic strategies and/or timely initiation of cardioprotective strategies.

## Author contributions

JJ, JP, and RP wrote the manuscript. JJ drew the figures and wrote the table. All authors read and approved the final manuscript.

## Abbreviations

AIC: anthracycline-induced cardiotoxicity
BNP: B-type natriuretic peptide
CAD: coronary artery disease
CT: computerized tomography
Dox: doxorubicin
^18^F-FDG: ^18^F-fluorodeoxyglucose
GLS: global longitudinal strain
H/M: heart-to-mediastinum
ID/g: injected dose/g
LV: left ventricle
LVEF: left ventricular ejection fraction
MRI: magnetic resonance imaging
MUGA: multigated acquisition
NLRP3: nucleotide-binding oligomerization domain (NOD-), leucine-rich repeat (LRR-), and pyrin domain (PYD)-containing protein 3
NT-proBNP: N-terminal proBNP
PET: positron emission tomography
PS: phosphatidylserine
ROS: reactive oxygen species
SPECT: single-photon emission computed tomography
TUNEL: terminal deoxynucleotidyltransferase-mediated dUTP nick-end labeling
WOR: washout rate.
